# Disagreeing on Whether Agreement Is Persuasive: Perceptions of Expert Group Decisions

**DOI:** 10.1371/journal.pone.0121426

**Published:** 2015-03-26

**Authors:** Ashley M. Votruba, Virginia S. Y. Kwan

**Affiliations:** 1 Department of Psychology, Arizona State University, Tempe, Arizona, United States of America; 2 Sandra Day O’Connor College of Law, Tempe, Arizona, United States, United States of America; Institut Pluridisciplinaire Hubert Curien, FRANCE

## Abstract

While expert groups often make recommendations on a range of non-controversial as well as controversial issues, little is known about how the level of expert consensus—the level of expert agreement—influences perceptions of the recommendations. This research illustrates that for non-controversial issues expert groups that exhibit high levels of agreement are more persuasive than expert groups that exhibit low levels of agreement. This effect is mediated by the perceived entitativity—the perceived cohesiveness or unification of the group—of the expert group. But for controversial issues, this effect is moderated by the perceivers’ implicit assumptions about the group composition. When perceivers are provided no information about a group supporting the Affordable Care Act—a highly controversial piece of U.S. legislation that is divided by political party throughout the country—higher levels of agreement are less persuasive than lower levels of agreement because participants assume there were more democrats and fewer republicans in the group. But when explicitly told that the group was half republicans and half democrats, higher levels of agreement are more persuasive.

## Introduction

In the United States, there are many issues on which the country is divided along the lines of the two primary political groups, the republicans and democrats, and public perceptions of decisions surrounding these issues are strongly influenced by the underlying political currents. For example, in 2010 President Barack Obama signed into law the controversial Affordable Care Act (ACA) [1] [2]. This act is one of the largest regulatory overhauls of the U.S. healthcare system and was intended to increase the number of Americans with health insurance. Although few Americans know, the ACA has a strong bipartisan foundation. The idea originated from a 1989 proposal by the conservative think tank the Heritage Foundation [3], it then became supported by democrats including President Obama [4], and the Senate committee that ultimately developed the bill consisted of both republicans and democrats [5]. Yet, most U.S. citizens perceive the ACA as being supported by democrats as part of a liberal agenda and fought by republicans. Would public perception of the ACA be different if Americans knew the bipartisan support for the ACA?

This article presents two studies examining how the composition of decision-making groups and their level of consensus, what we refer to as the level of “expert agreement”, influence perceivers in non-controversial and controversial contexts. The pilot study establishes the baseline influence of level of expert agreement when a decision is non-controversial. Then Study 1 ascertains the influences of the amount of expert agreement and information about group composition regarding a controversial ACA decision in which a legislative body is the decision-making group.

## Pilot Study

Compared to non-experts, experts: (1) are more persuasive; (2) elicit more change in attitudes; and (3) exert a stronger influence on behavioral compliance [6] [7] [8]. While the “expert” heuristic is documented for individual experts [9], we know little about the influence of groups of experts [7]. This leaves open the question of how messages from expert groups are perceived, especially for groups who are not in complete agreement. Research examining majority influences generally indicate that the more agreement there is for a position the more valid that position seems [10] [11]. This leads to the acceptance of the majority position and can induce significant attitude change [12] [13].

Also important to group perception is the psychological concept of entitativity, which is defined as “the degree of having the nature of an entity, of having real existence” [14]. High-entitativity groups are perceived as more unified compared to low-entitativity groups. When high-entitativity groups endorse a favorable position, perceiver processing is more superficial and heuristic resulting in more favorable perceptions of the argument [15]. Consequently, we expect that expert groups with higher levels of agreement would be perceived as having higher group entitativity, thus the recommendations presented by these expert groups would be perceived as more persuasive, compared to those presented by groups with lower levels of agreement. As a starting point, our pilot study focuses on non-controversial recommendations.

### Methods

#### Participants

Two-hundred and twenty four participants (58.5% women) completed this study for college course credits as part of a larger survey administered at the beginning of the semester. The average age was 19.01 years old. The participants were: 30.8% democrat, 26.8% republican, 26.8% independent, 8.0% libertarian, 0.9% green party, and 5.8% checked the “other” category. This study was approved by the Institutional Review Board at Arizona State University and participants provided written informed consent.

#### Design and Materials

Participants were randomly assigned to one of two conditions. First, participants received information providing them with background on the organization making the recommendation. This initial paragraph was where the level of expert agreement was manipulated. Those in the *high* agreement condition read, “An organization of health experts has made a recommendation to the public. The organization set up a panel of 10 experts to review the relevant research on the issue and vote in order to determine their recommendations. The following is their current recommendation which *10 out of 10* of the experts voted in favor of:. . .” For the *low* agreement condition, only the language in italics differed stating “6 out of 10”. After having reviewed the paragraph introducing the expert group making the recommendation, the participants then read the recommendation, which stated, “Our group of experts recommends reducing your consumption of sugar sweetened beverages, such as soda, to prevent an unhealthy weight.”

After having read one of two randomly assigned vignettes participants were asked, “How persuasive was this recommendation?” (rated on a scale of “1 = very unpersuasive” to “7 = very persuasive”). Then, to examine the influence of perceived entitativity, a single question measure of entitativity was used modeled after a measure developed by [15]. Participants were asked, “To what extent do the experts deciding on the recommendation qualify as a cohesive, unified group?” The response options ranged from “1 = Not Really a Group” to “7 = Very Much so a Group.”

### Results

**Fig 1:**
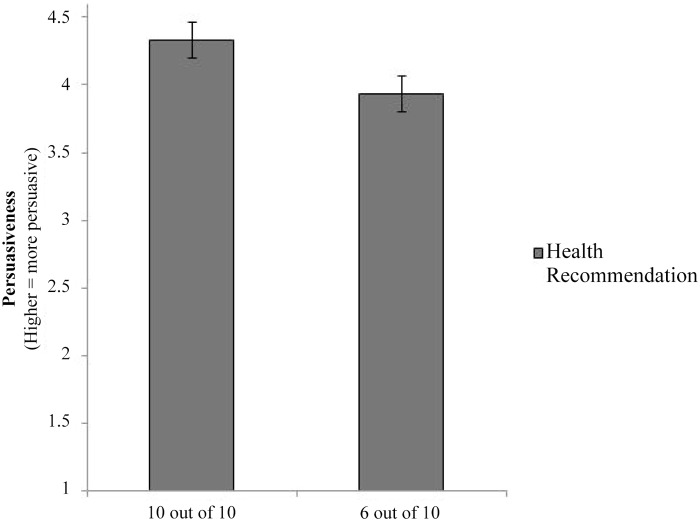
Pilot Study: Graph of means for Persuasiveness (“How persuasive was this recommendation?”).

Analyzing the data using a One-way ANOVA, a main effect of level of agreement was observed for the persuasiveness measure, *F*(1, 219) = 4.66, *p* = .032, η^2^ = .021. As predicted, recommendations from expert groups with high agreement were perceived as more persuasive than those with low agreement (see Fig. 1; 10 out of 10: *M* = 4.33, *SD* = 1.41; 6 out of 10: *M* = 3.93, *SD* = 1.39).

**Fig 2:**
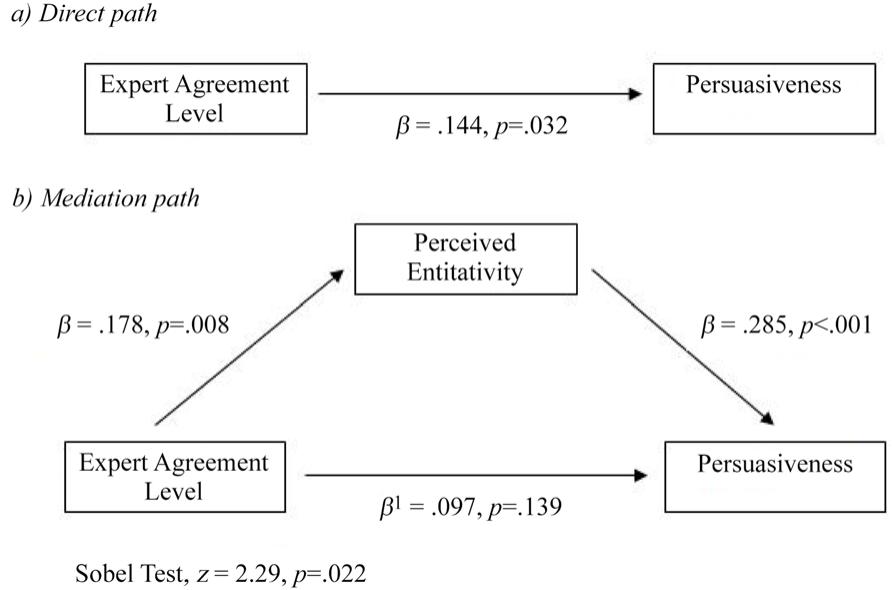
Pilot Study: Diagram of Entitativity Mediating the Relationship between the Level of Expert Agreement and Persuasiveness.

Next, we examined whether perceived entitativity mediated this effect using regression analyses. The results indicate that the level of agreement was associated with perceived entitativity. The more agreement among the experts, the more entitative the expert group making the recommendation seemed to the participants, *β* = .178, R^2^ = .032, *p* = .008 (see Fig. 2B: the mediation path). A separate regression including only the level of agreement in the model, as shown in Fig. 2A, showed that the level of agreement was associated with perceived persuasiveness. The higher the amount of agreement, the more persuasive the recommendation, *β* = .144, R^2^ = .021, *p* = .032. Perceived entitativity was also related to persuasiveness. The higher the perceived entitativity, the higher the score on the persuasiveness composite measure, *β* = .285, R^2^ = .081, *p* < .001. Finally, when the persuasiveness composite measure was regressed on perceived entitativity and the level of agreement, the perceived entitativity was a significant predictor of the persuasiveness of the recommendation (*β* = .268, *p*< .001) but the level of agreement was not (*β* = .097, *p* = .139; overall model R^2^ = .09).

This series of analyses demonstrates that the level of agreement did not remain a significant predictor of the persuasiveness of a recommendation when the perceived entitativity of the expert group making the recommendation was accounted for in the model. This suggests that the relationship between the level of agreement and the persuasiveness of a recommendation is mediated by perceived entitativity and is supported by the Sobel test, z = 2.29, *p* = .022. Participants in the high agreement condition perceived the expert group making the recommendation as having more entitativity and therefore found the recommendation more persuasive. Similarly, when an expert group making a recommendation has lower levels of agreement, then that group is perceived as having less entitativity and the recommendation is less persuasive.

## Study 1

A recent poll showed that 79% of democrats, compared to only 13% of republicans, supported the ACA [2]. Are higher levels of agreement still more influential for controversial topics, like the ACA, where support is divided by political affiliation? The answer may not be straightforward. People make implicit assumptions about decision-making groups. For controversial issues that divide along political party affiliation, perceptions of the groups’ decisions might be influenced by the perceived political bias of the group. When expert agreement is high and perceivers are uninformed about the groups composition, we expect participants to implicitly assume there is more of a skewed distribution of republicans and democrats—the direction of the skew depending on the outcome of the decision—leading them to view the decision less favorably. In contrast, when expert agreement is low, we expect participants to perceive the group as having closer to equal distributions of republicans and democrats, leading them to view the decisions more favorably. But if participants are informed that the expert group supporting the controversial issue was composed of half republicans and half democrats, then higher levels of expert agreement may be more influential. These hypotheses are in line with research on the multiple source effect. Harkins and Petty found that when participants were informed that there were multiple sources that formed a committee who were very similar, the persuasive advantage of having multiple courses presenting strong arguments was eliminated [16]. However, when participants were informed that the sources on the committee contained dissimilar perspectives, then the persuasive advantaged of multiple sources was present.

### Methods

#### Participants

Participants for this study were gathered using Amazon’s Mechanical Turk. In total, 287 participants (37.3% women) completed this 5-minute study for $ 0.50 in compensation for their time. The average age was 34.67 years old. The participants were: 39.7% democrat, 12.2% republican, 33.1% independent, 4.9% libertarian, 0.3% tea party, and 9.8% did not respond. This study was approved by the Institutional Review Board at Arizona State University and participants provided written informed consent.

#### Design and Materials

This study consisted of a 2 (level of expert agreement) x 2 (group composition) between-subjects design. The level of expert agreement was manipulated by telling participants that “9 out of 10” or “6 out of 10” experts voted in favor of the recommendation. We also manipulated information about the composition of the group by telling participants that the group consisted of 5 democrats and 5 republicans or providing them with no information about the group composition. This study was completed at the end of November 2013, which was shortly after the government shutdown because Republicans in the House and Senate had concerns about provision of the ACA in October 2013. Thus, this was a very controversial topic at the time, with which most Americans were familiar.

Participants first read the study materials, which provided some basic background on the ACA. Afterwards, participants read one of four paragraphs, which included the experimental manipulations. The paragraph for one of the conditions read, “Imagine a state legislature is meeting to decide whether to participate in one of the Affordable Care Act’s optional provisions. The legislature consists of 10 members *of which 5 are registered democrats and the other 5 are registered republicans*. They talked to many experts and looked at all available evidence as to whether participation would benefit the citizens of the state. After discussing the relevant issues, 6 out of 10 members of the legislature voted in favor of participating in the optional provision of the Affordable Care Act.” In this version, information is provided regarding the group composition, which is shown in italics. In the no information conditions, this information was omitted. The level of expert agreement was manipulated by changing the underlined text to “9 out of 10” for the high agreement conditions.

After having read the study materials, participants were then asked to answer the following questions in the order provided. To assess participants’ support for the decisions we asked, “To what extent do you support the decision made by the legislature?” Participants responded on a 7-point Likert scale ranging from “1 = Very Unsupportive” to “7 = Very Supportive.” In addition, participants were asked, “Assuming you are living in this state, how willing would you be to pay increased taxes to help support the optional provision of Affordable Care Act?” They responded on a 7-point Likert scale of “1 = Very Unwilling” to “7 = Very Willing.” Then, to assess our participants’ attitudes toward the ACA, we used a five-item scale that asked participants, “To what degree would you say that the Affordable Care Act optional provision is:” and then provided a series of 7-point Likert scale with the end points of: bad/good, harmful/beneficial, negative/positive, unnecessary/necessary, and foolish/wise. This scale had a Cronbach’s Alpha of. 977. Participants were also asked, “Of the experts in the group making the recommendation, how many do you think were democrats?” The same question was also asked for “republicans.”

### Results

**Fig 3:**
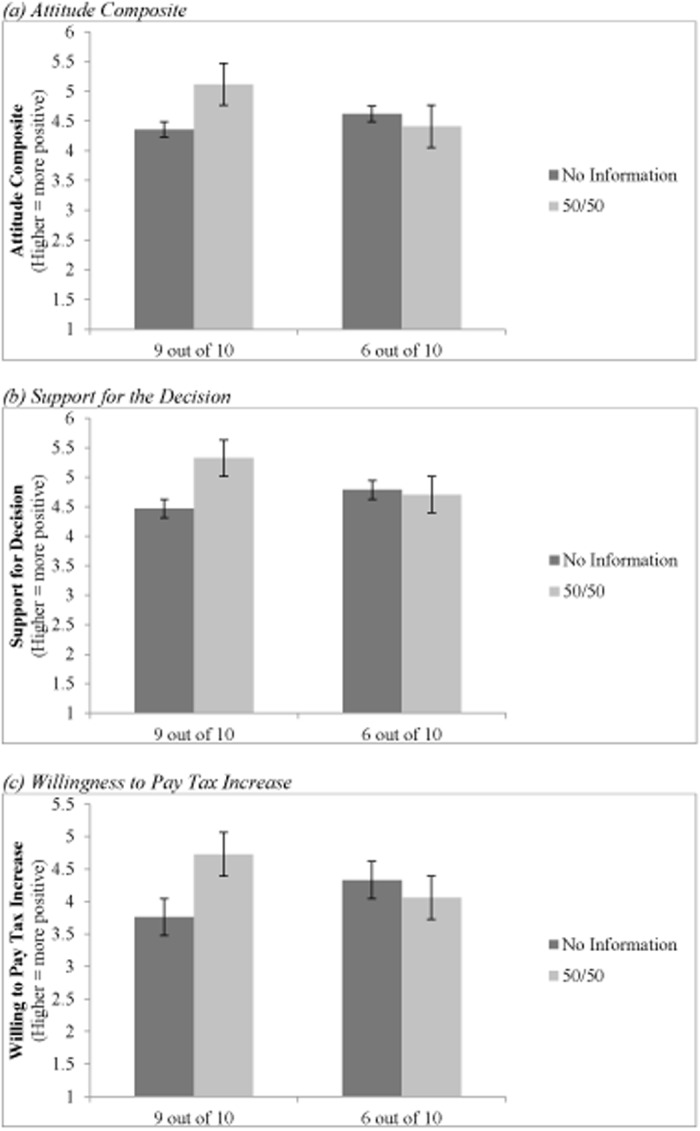
Study 1: Graph of means for (a) Attitude Composite, (b) Support of the Decisions, and (c) Willingness to Pay Increased Taxes.

Using an ANOVA to analyze the data, the results indicate a significant interaction between the level of expert agreement and the group composition for the attitude composite, *F*(1, 243) = 5.859, *p* = .016, η^2^ = .023, see Fig. 3A. Examining the simple effects for the no information conditions, there was no difference based on the level of agreement, *F*(1, 121) = .837, *p* = .362, η^2^ = .007. When the participants were not provided information regarding the number of democrats and republicans that were in the expert group, their attitudes toward the Affordable Care Act was similar when 9 out of 10 legislators voted in favor of the optional provision (*M* = 4.36, *SD* = 1.69) to when 6 out of 10 legislators voted in favor of the optional provision (*M* = 4.62, *SD* = 1.54). However, as predicted, when participants were told the legislature was half republicans and half democrats, higher levels of expert agreement were more persuasive than lower levels of agreement, *F*(1, 122) = 6.436, *p* = .012, η^2^ = .050 (9 out of 10 *M* = 5.12, *SD* = 1.56; 6 out of 10 *M* = 4.41, *SD* = 1.57).

We also found a significant interaction between the level of expert agreement and group composition in their willingness to support the decision, *F*(1, 257) = 5.204, *p* = .023, η^2^ = .020 (See Fig. 3B), and pay increased taxes, *F*(1, 258) = 7.356, *p* = .007, η^2^ = .028 (see Fig. 3C). The simple effects analyses showed when participants were provided no information about the group composition, there was no significant difference between the two levels of agreement in their willingness to support the decision, *F*(1, 128) = 1.117, *p* = .293, η^2^ = .009 (9 out of 10 *M* = 4.47, *SD* = 1.76; 6 out of 10 *M* = 4.79, *SD* = 1.63). Likewise there was also no difference in the participants’ willingness to pay increased taxes when the decision was supported by 6 out of 10 (*M* = 4.33, *SD* = 1.78) compared to when it was supported by 9 out of 10 legislators (*M* = 3.76, *SD* = 1.86), *F*(1, 129) = 3.192, *p* = .076, η^2^ = .024. This finding is consistent with the attitude composite findings indicating that for a controversial topic like the ACA, participants are not finding high levels of expert agreement more persuasive when they do not know the composition of the group.

Importantly, when participants were told the expert group consisted of half republicans and half democrats, the level of expert agreement had a significant effect on their support of the decision and willingness to pay increased taxes, Support: *F*(1, 129) = 4.798, *p* = .030, η^2^ = .036; Taxes: F(1,129) = 4.186, *p* = .043, η^2^ = .031. When 9 out of 10 legislators voted in favor of the optional provision more participants supported that decision (*M* = 5.33, *SD* = 1.56) and were willing to pay increased taxes (*M = 4*.*73*, *SD* = 1.71) than when 6 out of 10 voted in favor of it (Support: *M* = 4.71, *SD* = 1.71; Taxes: *M* = 4.06, *SD* = 2.02).

Next we tested our prediction that the assumed political affiliations of the group members varied across levels of expert agreement when participants are provided with no information about the group composition. Indeed, this was the case. As predicted, when participants were provided no information about the legislature’s composition, they thought there were significantly more democrats in the group in the 9 out of 10 condition (*M* = 6.92, *SD* = 2.29) compared to the 6 out of 10 condition (*M* = 5.61, *SD* = 1.33), *F*(1, 127) = 16.110, *p*< .001, η^2^ = .113. Participants also thought there were significantly fewer republicans in the 9 out of 10 condition (*M* = 3.19, *SD* = 2.38) compared to the 6 out of 10 condition (*M* = 4.15, *SD* = 1.27), *F*(1, 128) = 8.748, *p* = .004, η^2^ = .064. In addition, this effect held regardless of the political affiliation of the participant (political party and the level of agreement interaction was not significant, *F*(3, 121) = 1.076, *p* = .362). Thus, it seems that when there is high expert agreement in favor of the ACA, participants tend to perceive the legislature as having more democrat members and fewer republican members.

## Discussion

Our findings suggest that the level of expert agreement influences perceptions of the decision-making group’s message differently for controversial and non-controversial issues. For non-controversial recommendations, higher expert agreement is more persuasive and the effect is mediated by the perceived entitativity of the group. But when the issue is controversial and participants have no information about the group composition, high levels of expert agreement are no longer more influential and in some cases are even less influential. Our studies suggest this is the result of the perceivers’ implicit assumptions about the group composition. They assume that high agreement in support of the ACA indicates the decision-making group has more democrats and fewer republicans compared to when there is lower agreement. But when the group composition is known to be half republicans and half democrats, high expert agreement is once again more influential.

However, it is important to note that our conclusions regarding controversial and non-controversial issues are based on examining results from two different studies and our understanding of the effect could benefit from future research manipulating the controversial nature of the issue within the same study. Likewise, it should also be noted that the sample for Study 1 had a limited number of republican participants. Thus, the argument could be made that if our sample is skewed toward being democrat, then they may not perceive the ACA as a controversial issue. This could limit the interpretation of our findings and future research should conceptually replicate our findings with a more politically representative sample.

For public policy issues such as the ACA, even a small effect can have a profound influence [17] [18]. For example presidential elections are often won by only a couple of percentage points of the popular vote [19]. Although our effect sizes were relatively small, our studies only manipulated the level of expert agreement in a single short statement (“6 out of 10”) and yet the results still showed significant effects.

Provided the results of these studies, there are a number of different directions for future research. First, additional research could expand upon these findings by examining how the effects translate using behavioral measures. For example, after receiving the health recommendation from the Pilot Study researchers could examine whether there are any actual changes in health behavior. For example, the same recommendation from the Pilot Study could be used and research could offer participants a water or soda at the end of the study and note which beverage they choose. Based on our results from the pilot study, we predict that more participants would choose the water in the high expert agreement condition compared to the low expert agreement condition.

Another interesting direction for future research could examine how an individual’s initial opinion regarding a controversial topic affects their perceptions of the expert groups, their level of agreement on the issue, and their decision. It is possible that an individual’s initial attitudes could affect how they perceive the composition of an expert group when they are provided no information about the group. Finally, future studies might consider examining a pro-republican committee decision. In Study 1, we used a pro-democrat decision and have not yet examined whether the effect also holds for pro-republican decisions. A future study could examine the same controversial issue but manipulate whether the outcome of the committee’s decision favored the republican or democrat side of the issue.

Our findings have important implications. First, the main studies highlight the importance of perceivers’ implicit assumptions about a decision-making body for controversial issues. These assumptions influence how perceivers view the decisions. Second, the findings have interesting implications for understanding and influencing public opinion on public policy. As previous research has shown, an individual’s cultural beliefs about an issue can affect judgments of experts and their message [20]. Our findings expand this further and suggest that people perceive strong support for controversial issues amongst decision-making group members as indicating a biased group. But decisions from groups that are known to be bipartisan are viewed more favorably and policymakers could use this to increase public support for an issue.

## Supporting Information

S1 Dataset This is the dataset for the Pilot Study. (ZIP)

S2 Dataset This is the dataset for Study 1. (ZIP)
